# Exploring the potential of the ketogenic diet in managing metabolic syndrome: mechanisms, strategies, and future research directions

**DOI:** 10.3389/fnut.2025.1658691

**Published:** 2025-08-29

**Authors:** Jiping Chen, Jiawei Yao

**Affiliations:** ^1^School of Physical Education, Shandong University, Jinan, China; ^2^Culture and Tourism College, Guangdong Vocational Academy of Art, Foshan, China

**Keywords:** ketogenic diet, ketogenic, metabolic syndrome, mechanisms, strategies

## 1 Introduction

Metabolic syndrome (MetS) is a multifaceted condition characterized by a cluster of risk factors, including hypertension, elevated fasting blood glucose levels, increased waist circumference (WC), elevated triglyceride (TG) levels, and reduced high-density lipoprotein (HDL) cholesterol levels (1–5). Conventional treatments for MetS typically involve pharmacological interventions and physical activity (6, 7). The former, such as oral hypoglycemic and lipid-lowering drugs, are effective in controlling blood glucose and lipid levels but have some side effects and dependence (8, 9). The latter are helpful for weight control and improving insulin sensitivity, but these methods are often difficult to adhere to in the long term (10). Therefore, exploring more effective alternative treatment options has become an urgent need.

In recent years, the ketogenic diet (KD)—a low-carbohydrate, high-fat nutritional regimen—has attracted considerable attention (11). Specifically, KD promotes fat burning by increasing ketone body (KB) levels [e.g., β-hydroxybutyrate (β-BHB)], improves insulin sensitivity, regulates lipid metabolism, and reduces chronic low-grade inflammatory responses, which in turn alleviates the symptoms of MetS (12, 13). However, most current studies have focused on short-term effects in specific populations, leaving a gap in data from multicenter, large-scale clinical trials that encompass diverse ethnicities, age groups, and lifestyles (14–16). Future research should focus on the effects of the KD on various aspects of MetS, especially its adaptation in different populations. In addition, exploring optimized regimens, such as cyclical KD, and combining them with other therapeutic options may provide more effective avenues for the treatment of MetS.

## 2 KD strategies for MetS: based on a comprehensive review's perspective

MetS is a group of closely related metabolic abnormalities that typically include obesity, insulin resistance, hyperglycemia, and hyperlipidemia (17). Treatment for MetS does not focus on a single health indicator; instead, it aims to reduce the overall metabolic burden through comprehensive interventions (18). Sethi et al. (19) explored the effects of KD on metabolic health in patients with schizophrenia and bipolar disorder. Over 4 months, the KD led to a 12% reduction in body weight, a 12% decrease in body mass index (BMI), a 13% decrease in WC, and a 36% decrease in visceral fat. Furthermore, homeostatic model assessment for insulin resistance was reduced by 27%, TG levels decreased by 25%, and HDL levels increased by 2.7%. In a retrospective cohort study, Zachos et al. (20) assessed the metabolic health effects of low-fiber carbohydrates in patients with bipolar disorder. In contrast to the effects of KD, an increased intake of low-fiber carbohydrates was associated with an increased prevalence of MetS and higher BMI in this primary cohort. These findings suggest that the KD is highly effective in improving metabolic health, particularly in patients with psychiatric disorders.

Another 52-week study assessed the effects of an Asian KD (AKD) on individuals with MetS (16). Participants were randomly assigned to three groups: the whole egg intake AKD group (Yolk-AKD), the yolk-free AKD group (White-AKD), and a balanced low-caloric diet group (BLC). The results showed that the AKD group experienced significant improvements in weight, WC, and insulin sensitivity compared to the BLC group, with the Yolk-AKD group exhibiting the most pronounced weight loss. HDL cholesterol levels increased significantly in the AKD group, while TG levels decreased, indicating that AKD is efficacious in improving blood lipid profiles. Although low-density lipoprotein (LDL) increased, the improvement in HDL level helped to balance overall blood lipids. Furthermore, the AKD group exhibited lower levels of inflammation-related hormones, including a significant reduction in interleukin-6, tumor necrosis factor-alpha (TNF-α), and monocyte chemoattractant protein-1, underscoring its positive impact on metabolic health.

It is worth noting that a single dietary intervention has obvious limitations in understanding the comparative analysis of the KD with other dietary interventions, especially in the context of MetS. Therefore, in order to show the differences and advantages of the KD vs. other dietary regimens in improving various aspects of MetS, Castaldo et al. (14) investigated the effects of combining a KD with a Mediterranean diet for the treatment of obesity. The results showed that the KD resulted in significant reductions in body weight and abdominal fat, along with improvements in blood glucose levels, lipid profiles, and liver function. While the Mediterranean diet phase also improved metabolic health to some extent compared to KD, a rebound in blood glucose and lipid levels was observed, indicating lower resilience. Overall, the combined approach of the ketogenic and Mediterranean diets proved highly effective in enhancing body weight reduction and metabolic health and lowering cardiovascular risks in patients with obesity. Subsequently, Genco et al. (21) examined the impact of combining a very low-calorie KD with an intragastric balloon (Orbera) on weight loss outcomes in patients with obesity. The study included 80 patients with obesity who were randomly assigned to two groups after 4 months from the start of the study: one group followed a KD with an intragastric balloon (Group A), while the other group adhered to a low-calorie diet (LCD) with an intragastric balloon (Group B). The results revealed that Group A experienced significantly greater weight loss than Group B (8 kg vs. 3 kg), with a total weight loss of 19 kg in Group A compared to 12 kg in Group B (*p* < 0.05). This study demonstrated that an intervention program combining a KD with an intragastric balloon not only enhances weight loss but also has important clinical significance in improving various metabolic indices in MetS, suggesting its potential in the comprehensive management of MetS.

In addition, Ghorbanian et al. (15) investigated the effects of a KD and aerobic exercise (AE) on the metabolic health of middle-aged men with MetS. The results revealed that the AE+KD group experienced significant reductions in body weight, BMI, and body fat percentage, alongside substantial decreases in retinol binding protein 4 levels. The AE intervention also led to significant reductions in fatty acid binding protein 5 levels. Furthermore, the AE+KD group demonstrated notable improvements in insulin resistance and increased insulin sensitivity.

## 3 Potential mechanisms of KD on MetS

Recent studies have shown that dietary patterns with increased protein intake trigger the release of anorexigenic hormones, such as glucagon-like peptide-1 (GLP-1), cholecystokinin (CCK), and peptide YY (PYY), which collectively contribute to reduced appetite. This mechanism is particularly relevant in the management of MetS, where appetite control is essential for weight management. Hall et al. (22) examined the effects of casein and whey proteins on appetite and gastrointestinal hormone secretion. Their findings revealed that whey protein notably elevated plasma amino acid levels, stimulating the secretion of CCK and GLP-1, thereby enhancing satiety. In a similar vein, Lejeune et al. (23) investigated the impact of a high-protein (HP) diet vs. an adequate-protein diet on 24-h satiety, energy expenditure, and substrate metabolism. Although energy intake did not differ substantially between the two diets, energy expenditure and fat oxidation were elevated in the HP diet, suggesting that HP diets may play a crucial role in improving insulin sensitivity and long-term weight management, thereby addressing multiple aspects of MetS.

KD influences blood pressure through the renin-angiotensin-aldosterone (RAA) system. For instance, Belany et al. (24) investigated the effects of a low-calorie, low-fat diet (LFD) compared to a KD on the human body, specifically focusing on aldosterone and renin. The study revealed that after 6 weeks of intervention, aldosterone levels increased significantly in the KD group, while no notable changes were observed in the LFD group. Specifically, aldosterone levels in the KD group increased by 88% and 144%, which may be attributed to the elevated concentration of KBs. Moreover, despite the rise in aldosterone, cardiovascular metabolic risks, including blood pressure and blood glucose levels, remained unaffected in the KD group. Despite elevated aldosterone, blood pressure, and blood glucose levels were not adversely affected in the KD group, suggesting that the KD may have a potentially protective role in the management of MetS, particularly for blood pressure and cardiovascular health.

Evidence from patients with type 2 diabetes mellitus (T2DM) indicates that the stringent restriction of carbohydrates in a KD diminishes the intestinal absorption of monosaccharides, resulting in lowered blood glucose levels and minimized glycemic fluctuations. The trial conducted by Yancy et al. (25) assessed the effects of a low-carbohydrate KD in overweight individuals with T2DM over a 16-week period. The findings revealed substantial improvements in weight, WC, diastolic blood pressure, and glycemic control, as evidenced by reductions in glycated hemoglobin levels. In their review, Bolla et al. (26) explored the role of low-carbohydrate and KDs in managing both type 1 and type 2 diabetes. This finding demonstrates the potential of the KD in the short-term management of MetS, but more clinical studies are needed to validate its long-term effects and safety.

In addition to this, MetS is usually accompanied by chronic low-grade systemic inflammation that is closely associated with MetS features such as insulin resistance, abdominal fat accumulation, and dyslipidemia (27). It has been found that one of the main mechanisms by which the KD modulates inflammation is by promoting an increase in the level of circulating β-BHB, the primary KB, which can inhibit the inflammatory response through multiple pathways (28). β-BHB inhibits the expression of pro-inflammatory genes (e.g., TNF-α, IL-1β, and NF-κB) by upregulating anti-inflammatory genes, such as NF-κBIA and MAP3K8, thereby reducing the release of inflammatory factors (28–32). In addition, KD directly affects lipid profile and insulin sensitivity by limiting the intake of digestible carbohydrates. Specific mechanisms include decreasing insulin secretion, promoting lipolysis, and increasing KB levels, thereby improving insulin signaling (33, 34). Through these mechanisms, the KD not only helps alleviate inflammation associated with MetS but also significantly improves metabolic indices, such as body weight, blood glucose, blood lipids, and blood pressure, thus providing an important adjunctive role in the treatment of MetS.

## 4 Discussion

KD, characterized by its low-carbohydrate and high-fat composition, has demonstrated considerable potential in enhancing metabolic health, particularly in areas such as weight management, insulin sensitivity, and lipid regulation (Table 1). Nonetheless, there is ongoing debate regarding whether the KD surpasses traditional dietary approaches in terms of adherence, nutritional adequacy, and lifestyle compatibility. Other dietary approaches, such as the Mediterranean diet, have also shown substantial effectiveness in mitigating the symptoms of MetS, offering potentially greater feasibility for long-term adherence (35, 36). In contrast, the prolonged use of the KD may carry certain risks, particularly regarding nutritional deficiencies and cardiovascular health. Chronic carbohydrate restriction may result in deficiencies of essential micronutrients, such as vitamin C, magnesium, and calcium, thereby increasing the likelihood of diminished bone density and compromised immune function. Therefore, it is crucial to compare the long-term effects of these dietary strategies, with particular attention to adherence and nutritional balance, to guide future research. Specifically, future studies should examine the sustained health outcomes of the KD for individuals with MetS, comparing its safety profile to other dietary alternatives.

**Table 1:** Characteristics of included studies.

**Study**	**Study design**	**Cases**	**Characteristics of KD**	**Components of metabolic syndrome**
Castaldo et al. (14)	Obs Study	73	Consume a very low-calorie (< 500 kcal/day) protein-based diet providing approximately 10–20g of carbohydrates and lipids per day.	BP: reduced (SBP: *p* < 0.001, DBP: p < 0.001 during OD); TGs: reduced (*p* < 0.001 for both OD and hypo-MD phases); HDL: decreased during OD (*p* < 0.001) but increased during hypo-MD (*p* < 0.001); LDL: reduced (*p* < 0.001 during OD phase, stable during hypo-MD phase); WC: reduced (*p* < 0.001 for both OD and hypo-MD phases).
Genco et al. (21)	RCT	80	< 800 kcal diet structured with protein 1.2 ± 0.2 g/day of ideal weight and carbohydrates < 50 g/day; lipids >10–15 g/day, supported with vitamins and minerals.	SBP: reduced (*p* < 0.05); TGs: reduced (*p* < 0.01); HDL: increased (*p* < 0.05); LDL: reduced (*p* < 0.05); WC: reduced (*p* < 0.05).
Ghorbanian et al. (15)	RCT	10	Based on a 2,900 kcal calorie intake, but limited to 30–40 g/day of carbohydrate intake.	Blood glucose: reduced (*p* < 0.001); SBP: reduced (*p* < 0.005); TGs: reduced (*p* < 0.02); HDL: increased (not statistically significant); LDL: increased (*p* < 0.02); WC: reduced (*p* < 0.001).
Pinsawas et al. (16)	RCT	54	Reduction in carbohydrate intake to < 50 g/day and an emphasis on a low-saturated fat diet.	Blood glucose: reduced (*p* < 0.05 for White-AKD group at 6 weeks); SBP: reduced (significant decrease for White-AKD at weeks 35–52, *p* < 0.05); TGs: reduced (significant reduction at weeks 12 and 35 for White-AKD and Yolk-AKD groups, *p* < 0.05); HDL cholesterol: increased (significant increase for White-AKD group at 35–52 weeks and Yolk-AKD at 52 weeks, *p* < 0.05); LDL cholesterol: increased (significant increase at 35 weeks for AKD groups, *p* < 0.05); WC: reduced (significant reduction for both AKD groups, *p* < 0.05).
Sethi et al. (19)	PT	23	Macronutrient proportion 10% carbohydrate, 30% protein, and 60% fat.	Blood glucose: reduced by 3.6% (*p* < 0.001); SBP: reduced by 6.4% (*p* < 0.005); TGs: reduced by 20% (*p* < 0.02); HDL: increased by 2.7% (*p* > 0.05); LDL: increased by 21% (*p* < 0.02); WC: reduced by 11% (*p* < 0.001).

RCT, randomized controlled trial; PT, pilot trial; Obs Study, observational study; BP, blood pressure; SBP, systolic blood pressure; DBP, diastolic blood pressure; TGs, triglycerides; HDL, high-density lipoprotein; LDL, low-density lipoprotein; WC, waist circumference; AKD, Asian ketogenic diet; White-AKD, yolk-free AKD; Yolk-AKD, the whole egg intake AKD.

Beyond clinical research, recent advancements in molecular biology and epidemiology have provided new insights into the underlying mechanisms of the KD. For instance, studies indicate that the KD may play a critical role in reducing inflammation and enhancing insulin sensitivity by modulating gut microbiota, activating KB receptors, and influencing fatty acid metabolism pathways (37, 38). Additionally, emerging research suggests that the KD may facilitate fat oxidation and weight regulation by optimizing energy metabolism in liver and muscle cells (39, 40). These molecular findings open valuable avenues for future research, particularly in refining the KD approach to minimize adverse effects while maximizing therapeutic outcomes.

Given existing research gaps, several pertinent questions and hypotheses for future investigation into the KD's effects on MetS arise: (1) a comparative analysis of the KD vs. other low-carbohydrate diets in terms of long-term adherence, metabolic health, and cardiovascular risks; (2) the effects of prolonged KD on micronutrient deficiencies, bone health, and cardiovascular function; and (3) the mechanisms by which the KD influences gut microbiota and metabolic pathways in the management of MetS.
